# Supplementary material for: A drought stress-responsive metabolite malate modulates stomatal responses through G-protein-dependent pathway in grapevine and Arabidopsis
Source: Mol Hortic. 2026 Jan 6;6:2. doi: 10.1186/s43897-025-00181-z (PMC12772033; doi:10.1186/s43897-025-00181-z)
Supplement: Supplementary file 1 — Supplementary Material 1: Supplementary Figure S1. Metabolome analysis in grapevine leaves during dehydration treatment. Supplementary Figure S2. Ca2+ response to acetate in guard cells. Supplementary Figure S3. VvSLAC1 activity in the presence of TCA cycle metabolites. Supplementary Figure S4. Malate-induced stomatal closure in the presence of inhibitors. Supplementary Figure S5. ROS production in the presence of TCA cycle metabolites. [file 43897_2025_181_MOESM1_ESM.docx]

**Supplementary data for**

**A drought stress-responsive metabolite malate modulates stomatal responses through G-protein-dependent pathway in grapevine and Arabidopsis**

Yoshiharu Mimata^1^, Ruhai Gong^1,2^, Xuanxuan Pei^1^, Guochen Qin^1^, Wenxiu Ye^1*^

^1^Peking University Institute of Advanced Agricultural Sciences, Shandong Laboratory of Advanced Agricultural Sciences in Weifang, Shandong 261325, China

^2^College of Horticulture, Shanxi Agricultural University, Shanxi 030801, China

*Corresponding author: wenxiu.ye@pku-iaas.edu.cn

This file includes Supplementary Figure S1–5 and Supplementary Table S2–3.


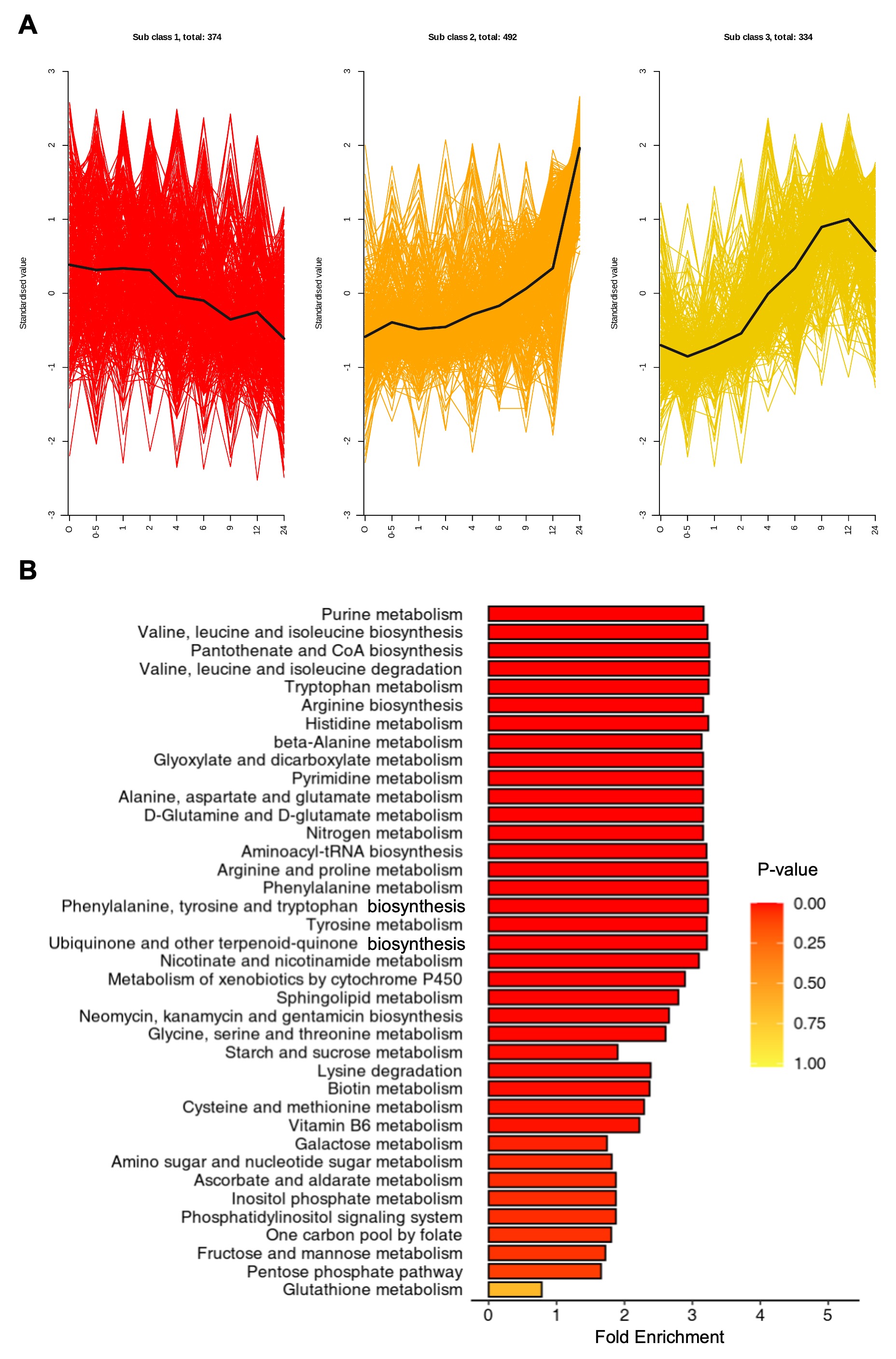


**Supplementary Figure S1. Metabolome analysis in grapevine leaves during dehydration treatment.**

**A)** K-Means plot of differential metabolite. Black lines indicate the means in each group. The ordinate represents the standardized relative content of metabolites. Sub class represents the metabolite category number with the same changing trend, with total: # indicating the number of metabolites in this category.

**B)** MSEA of differential metabolites. Bar color indicates the significance of difference, and bar lengths represent the fold enrichment. Significance was determined by hypergeometric test’s P-values.

**Supplementary Figure S2. Ca^2+^ response to acetate in guard cells.**

Representative traces of fluorescence emission intensity in *A. thaliana* guard cells expressing the Ca^2+^ sensor Yellow Cameleon 3.6. Grey bars indicate the time point when treatment was applied.

**Supplementary Figure S3.** VvSLAC1 activity in the presence of TCA cycle metabolites.

**A)** Representative whole-cell negative current traces during perfusion with TCA cycle metabolites in Xenopus oocytes expressing VvSLAC1. The whole-cell negative current recording in the presence of external malate showed as red trace. The voltage pulse was commanded to clamp the membrane potential from +60 mV to −120 mV in ramp for 1.8 seconds with a holding potential of 0 mV.

**B)** Average steady-state negative currents at −120 mV. Data are the mean ± SE (n = 6). Different letters indicate statistical significances within condition based on one-way ANOVA with Tukey’s HSD test, P < 0.05. Abbreviations: Suc, succinate; Fum, fumarate; Mal, malate; Oxal, oxalacetate; Keto, α-ketoglutarate; Cit, citrate; Isocit, isocitrate; cis-Aco, cis-aconitate; Ace, acetate; Pyr, pyruvate.


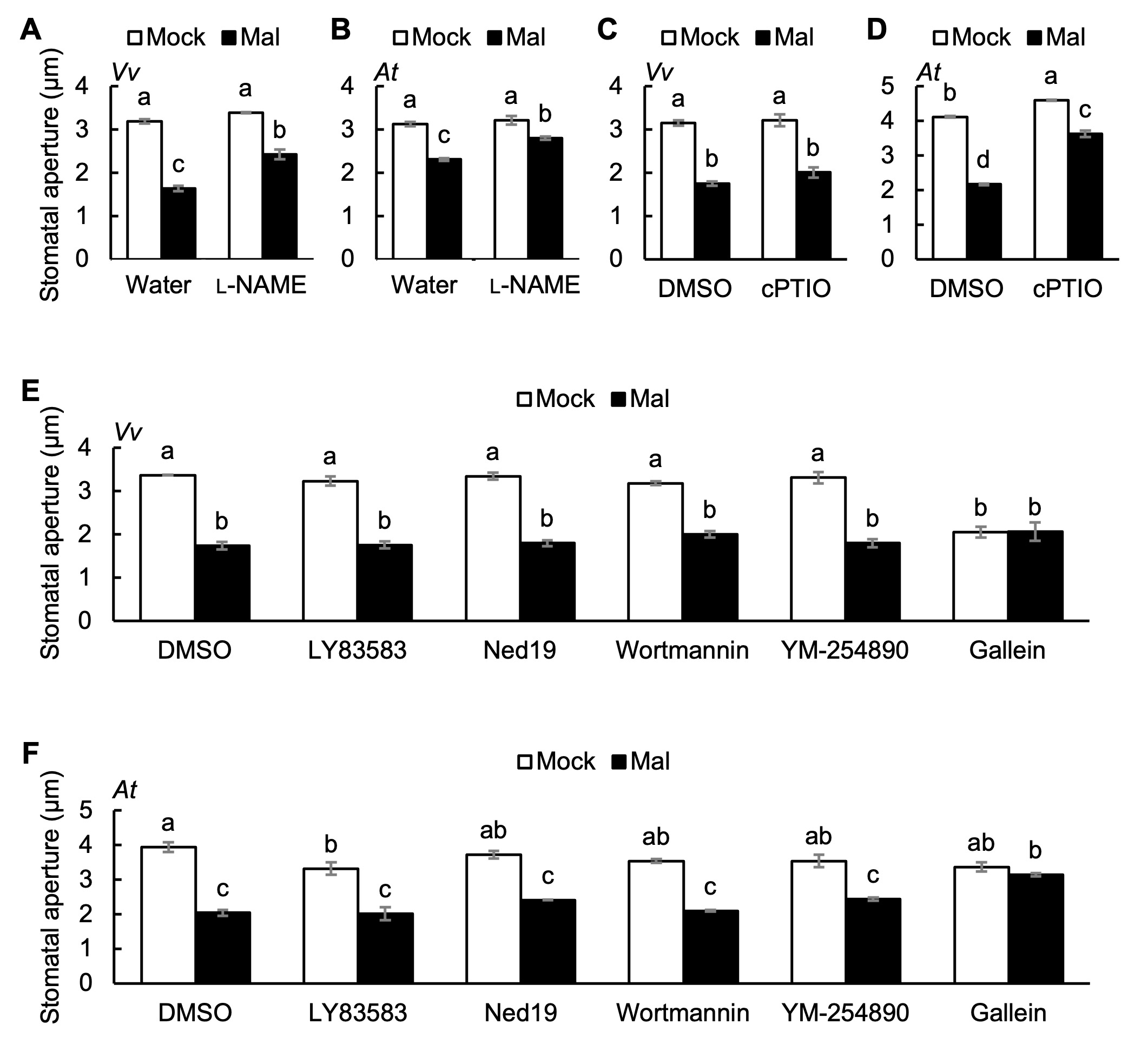


**Supplementary Figure S4. Malate-induced stomatal closure in the presence of inhibitors.**

**A to F)** Effects of inhibitors on malate-induced stomatal closure in **A, C and E)** *V. vinifera* or **B, D and F)** *A. thaliana* leaves. Averages of stomatal apertures from four independent experiments (n = 4) are shown. Data are the mean ± SE. Different letters indicate statistical significances based on two-way ANOVA with Tukey’s HSD test, P < 0.05.

**Supplementary Figure S5.** **ROS production in the presence of TCA cycle metabolites.**

**A)** Effects of TCA cycle metabolites on ROS production in *A. thaliana* guard cells.

**B)** Effects of La^3+^ on malate-induced ROS production in *A. thaliana* guard cells. The ROS-sensitive dye, 2',7'-dichlorodihydrofluorescein diacetate (H_2_DCF-DA) was used for ROS detection in guard cells. Fluorescence intensity was normalized to mock value **A)** or mock value in water **B)**. Averages from three independent experiments (n = 3) are shown. Data are the mean ± SE. Different letters indicate statistical significances based on one-way ANOVA with Tukey’s HSD test **A)** or two-way ANOVA with Tukey’s HSD test **B)**, P < 0.05.

**Supplementary Table S2. List of inhibitors used in this work.**

| **Name** | **Inhibitor type** | **Final concentration** |
| --- | --- | --- |
| DIDS | Anion channel blocker | 100 μM |
| 9-AC | Anion channel blocker | 100 μM |
| BAPTA | Extracellular Ca^2+^ chelator | 100 μM |
| Nifedipine | Ca^2+^ channel blocker | 10 μM |
| La^3+^ (LaCl_3_) | Ca^2+^ channel blocker | 1 mM |
| Nicotinamide | cADPR synthesis inhibitor | 50 mM |
| Alloxan | cAMP synthesis inhibitor | 1 mM |
| Neomycin | IP_3_ synthesis inhibitor | 50 μM |
| SHAM | Peroxidase-catalyzed ROS production inhibitor | 2 mM |
| l-NAME | NO synthetase inhibitor | 25 μM |
| cPTIO | NO scavenger | 100 μM |
| LY83583 | NO-sensitive guanylate cyclase inhibitor | 2 μM |
| Ned 19 | NAADP antagonist | 10 μM |
| Wortmannin | PIP_3_ synthesis inhibitor | 5 μM |
| YM-254890 | G-protein inhibitor | 10 μM |
| Gallein | G-protein inhibitor | 10 μM |
| GDPβS | G-protein inhibitor | 10 μM |
| GRK2i | G-protein inhibitor | 5 μM |
| Suramin | G-protein inhibitor | 100 μM |

**Supplementary Table S3. List of primers used in this work.**

| **Name** | **Sequence** |
| --- | --- |
| VvSLAC1_pNB1u_F | aaacctcagcgaattcATGGACAGAAGACCGACTTC |
| VvSLAC1_pNB1u_R | gcctattcccaagcttTCAGTGCTCTGCTTCCTTC |
| VvSLAC1F440A_F | TACACCgctCCCATGACAACAGTATCAGTGGC |
| VvSLAC1F440A_R | CATGGGagcGGTGTAAGACCACCATGCCAC |
